# Efficacy of Ligustrazine Injection as Adjunctive Therapy in Treating Acute Cerebral Infarction: A Systematic Review and Meta-Analysis

**DOI:** 10.3389/fphar.2021.761722

**Published:** 2021-11-22

**Authors:** Huikai Shao, Xia He, Lijuan Zhang, Shan Du, Xiaoqing Yi, Xiaojiao Cui, Xinxia Liu, Shengfeng Huang, Rongsheng Tong

**Affiliations:** ^1^ Department of Pharmacy, Sichuan Academy of Medical Sciences and Sichuan Provincial People’s Hospital, Chengdu, China; ^2^ Personalized Drug Therapy Key Laboratory of Sichuan Province, School of Medicine, University of Electronic Science and Technology of China, Chengdu, China; ^3^ Key Laboratory of Molecular Target and Clinical Pharmacology and the State and NMPA Key Laboratory, School of Pharmaceutical Sciences and the Fifth Affiliated Hospital, Guangzhou Medical University, Guangzhou, China

**Keywords:** ligustrazine injection, adjunctive therapy, acute cerebral infarction, systematic review, meta-analysis

## Abstract

**Background:** Ligustrazine injection has been widely used as adjunctive therapy in the treatment of acute cerebral infarction (ACI) during the past decades in China, but its clinical efficacy is not yet well confirmed. This study aims to evaluate the efficacy of ligustrazine injection as adjunctive therapy for ACI.

**Methods:** Databases including China National Knowledge Infrastructure (CNKI), China Science and Technology Journal Database (VIP), PubMed, Medline, Google Scholar, Chinese Biomedical Literature Database, Cochrane Library, Embase, Sino-Med, Wanfang Database, and Chinese Science Citation Database were systematically searched for the published randomized controlled trials (RCTs) on ligustrazine injection in the treatment of ACI until November 2020. Meta-analysis was performed on the primary outcome measure (i.e., clinical effective rate) and the secondary outcome measure [i.e., neurological deficit score (NDS), fibrinogen, low shear blood viscosity (LBV), and high shear blood viscosity (HBV)]. The quality of the included RCTs was assessed according to the M scoring system (the refined Jadad scale). Sensitivity analysis and subgroup analysis were conducted according to the methodological quality, years of publication, and sample size.

**Results:** Nineteen RCTs, containing 2022 patients, were included in this study. Meta-analysis indicated that ligustrazine injection combined with Western medicine could achieve a better effect in the treatment of ACI than using Western medicine alone in terms of clinical effective rate (RR = 1.24; 95% CI, 1.19–1.29), NDS (MD = −3.88; 95%CI, −4.51 to −3.61), fibrinogen (MD = −0.59; 95% CI, −0.76 to −0.42), LBV (MD = −2.11; 95% CI, −3.16 to −1.06), and HBV (MD = −0.88; 95% CI, −1.20 to −0.55).

**Conclusions:** This research indicated that ligustrazine injection as adjunctive therapy seemed to be more effective than using western medicine alone in treating ACI. However, more evidence is required to confirm the efficacy of ligustrazine injection due to the low methodological quality of the included RCTs.

## Introduction

Acute cerebral infarction (ACI), a neurological deficit syndrome caused by circulatory dysfunction, is a major disease leading to disability or death. It was estimated that about 6.17 million people died from ACI in 2017 in the world and there is still no effective way to reduce the mortality (Inoue et al., 2006; Johnston et al., 2009; GBD 2016 Stroke Collaborators, 2019). At present, conventional therapy for treating ACI mainly contains anticoagulants, antithrombotics, and thrombolytics (Powers et al., 2018). As the most important therapy in the treatment of ACI, recombinant tissue plasminogen activator (rt-PA) and urokinase (United Kingdom) are the frequently used thrombolytic agents in China (The fourth session of the National Cerebrovascular Conference, 1996). However, the success of thrombolytic treatment is determined by the strict time window (within 4.5 h) and less than 3% of ACI patients can benefit from it (Asadi et al., 2015). Reasonable use of anticoagulation and antiplatelet agents can improve symptoms and reduce the recurrence rate for ACI patients in some degree, but the risk of intracranial hemorrhage may increase with their use [CAST (Chinese Acute Stroke Trial) Collaborative Group, 1997; International Stroke Trial Collaborative Group, 1997; Powers et al., 2018]. Although these Western medicines can accelerate a patient’s recovery to a certain extent, drug side effects and resistances are accompanied by their wide application in clinical practice [CAST (Chinese Acute Stroke Trial) Collaborative Group, 1997; International Stroke Trial Collaborative Group, 1997]. Traditional Chinese medicine (TCM) combined with conventional Western medicine could be a candidate to overcome the above shortcomings because of the remarkable effectiveness, high bioavailability, and rapid action (Yu et al., 2019).

As the dried rhizome of *Conioselinum anthriscoides* “Chuanxiong”, Chuanxiong (Conioselinum anthriscoides (H.Boissieu) Pimenov & Kljuykov cult.) was first recorded in Shennong’s Classic of Materia Medica (the earliest Pharmacopoeia of China) from the Warring States Period to the Han Dynasty (Zheng et al., 2018). In the past thousands of years, this herbal medicine has been widely used in treating cerebrovascular, cardiovascular, and renal diseases (Zhao et al., 2016). Ligustrazine (2, 3, 5, 6-tetramethylpyrazine, Supplementary Figure S1) is the active ingredient of *Chuanxiong Rhizome* and it was first isolated in 1957. Ligustrazine injection has been widely used for the treatment of ACI (Gao et al., 2015) and coronary heart disease (Guo et al., 2016) among the physicians in China during the past decades (Shao et al., 2015). Owing to the impressive effects of antithrombosis, the inhibition of platelet aggregation, and protection of endothelium, ligustrazine injection can improve the neurological function, blood flow, and attenuate cerebral ischemia reperfusion injury for ACI patients (Yu et al., 2016). To provide better efficacy in the treatment of ACI, ligustrazine injection is usually combined with Western medicine (Ozagrel sodium injection, edaravone injection, citicoline sodium injection, aspirin, alteplase, etc.) in China.

At present, a large number of clinical studies on ligustrazine injection as adjunctive therapy for ACI have been reported with potential positive results. However, neither systematic review nor meta-analysis on the efficacy of ligustrazine injection combined with Western medicine in the treatment of ACI is reported until now. Herein, this study aims to conduct a comprehensive and PRISMA-compliant systematic review with sensitivity and subgroup analysis to confirm the clinical efficacy of ligustrazine injection as adjunctive therapy in treating ACI (Moher et al., 2009).

## Methods

### Eligibility Criteria

Two authors independently searched and selected the eligible studies based on the following criteria: (1) Randomized controlled trials (RCTs) concerning the efficacy of ligustrazine injection as adjunctive therapy (ligustrazine injection combined with Western medicine vs*.* Western medicine alone) for the treatment of ACI were included in this study. The type of Western medicine used in the control group could be classified into antiplatelet drugs (ozagrel sodium injection, aspirin, clopidogrel, venorruton), anticoagulants (low-molecular-weight heparins, calcium injection), thrombolytic drugs (alteplase injection, lumbrokinase), fibrinolytic drugs (fibrinogenase injection), neuroprotectants (edaravone, citicoline sodium injection, nimodipine), statins (simvastatin, stabilizing atherosclerotic plaque), and dehydration drugs (mannitol injection, relieving cerebral edema); (2) duration of intervention was at least 1 week; (3) sample size of the included RCTs was at least 40 participants; (4) the ACI patients should meet the diagnostic criteria according to the standards revised by the fourth National Conference on Cerebrovascular Diseases in 1995 (Chinese society for Neuroscience, 1996); (5) primary outcome measure was the clinical effective rate, calculated with the following equation (Zhang et al., 2015): clinical effective rate (%) = (number of recovered patients + number of patients with significant improvement + number of patients with improvement)/total number of patients × 100%. The reduction of neurological deficit score (NDS) was determined as efficacy criteria. Recovery was determined that NDS was reduced from 91 to 100%. Significant improvement was determined that NDS was reduced from 46 to 90%. Improvement was determined that NDS was reduced from 18 to 45%. Ineffective was determined that NDS was reduced to less than 17%. Secondary outcome measures included NDS, fibrinogen, low shear blood viscosity (LBV), and high shear blood viscosity (HBV).

RCTs were excluded when they did not satisfy the above criteria and: (1) Publication date of studies was not between the years 2007 and 2019; (2) formulation and dosages of intervention in the treatment group or control group were not specifically provided in the RCTs; (3) duplicate publication or incomplete data was found; (4) any other Chinese medicine was involved in the treatment group or control group; and (5) object of RCTs was animal or tissue cell.

### Information Sources

The following databases were systematically searched by two authors for the published RCTs on the adjunctive therapy of ligustrazine injection for ACI during the years between 2007 and 2019, including China National Knowledge Infrastructure (CNKI), China Science and Technology Journal Database (VIP), PubMed, Medline, Google Scholar, Chinese Biomedical Literature Database, Cochrane Library, Embase, Sino-Med, Wanfang Database, and Chinese Science Citation Database. The latest search for the databases was performed on November 1, 2020.

### Search Strategies

For English databases, the following keywords were searched in separate or joint ways: ligustrazine, tetramethylpyrazine, ligustrazine injection, acute cerebral infarction, cerebral infarction, stroke, and ischemic stroke. For Chinese databases, the following keywords were searched in separate or joint ways: Chuanxiongqin zhusheye (ligustrazine injection); Chuanxiongqin (ligustrazine, tetramethylpyrazine); Jixingnaogengsi (acute cerebral infarction); Naogengsi (cerebral infarction); Zhongfeng (stroke); and Quexuexingcuzhong (ischemic stroke). The detailed search strategy is shown in the Supplementary Text S1. Moreover, the references listed in the selected studies were searched to obtain the additional RCTs.

### Study Selection

Basing on the inclusion and exclusion criteria, two authors independently searched the databases for eligible RCTs. Disagreements between the two authors were solved by discussion.

### Data Collection Process

`One author screened the full text of the eligible RCTs and collected data. Another author carefully checked the accuracy and completeness of the collected data. Disagreements during this process were solved through discussion with the third author. Review Manager 5.2 (Cochrane Collaboration, Nordic Cochrane Centre, Copenhagen, Denmark) and STATA 12.0 (Stata Corp LLC, Texas, United States) were employed to evaluate the collected data in this meta-analysis.

### Data Items

The following items in each study were collected: (1) First author, years of publication, and country of the included RCTs; (2) sample size of ACI patients; (3) therapeutic intervention (dosages and duration) in the treatment and control group; (4) outcome measures; and (5) adverse reactions.

### Risk of Bias in Individual Studies

Two authors independently evaluated the quality of the included RCTs basing on the items of randomization methods, concealment of allocation, and blinding in the Jadad score (Supplementary Table S1) and M scoring system (Supplementary Table S2) (Jadad et al., 1996; Shao et al., 2018).

### Quality Assessment

Jadad score was employed to assess the included RCTs basing on the description of randomization, blinding, and withdrawals (dropouts), and it ranges from 0 (poorest) to 5 (highest). Scores were obtained if randomization was described in the included RCTs, 1 score; appropriate randomization method, 1 score; double-blinding was described, 1 score; appropriate double-blinding method, 1 score; withdrawals and dropouts were described in the RCT, 1 score. RCTs scored 1 or 2 scores were regarded as low quality and 3 to 5 scores were regarded as high quality. Two authors independently evaluated the methodological quality of the included RCTs according to the Jadad score and M scoring system (range from −1 to 7 scores, RCTs with M score >3 were considered as high quality). Any controversy during the quality evaluation between the two authors was resolved by discussion.

### Sensitivity and Subgroup Analysis

In this study, sensitivity analysis was conducted to evaluate the influence of low quality RCTs on the overall efficacy of ligustrazine injection combined with Western medicine in the treatment of ACI. Based on the sample size and years of publication of the included RCTs, subgroup analysis was conducted to evaluate if the overall effects were homogeneous in subgroups.

### Publication Bias

In order to evaluate the potential publication bias, the funnel plots generated by STATA 12.0 were adopted in this meta-analysis. Begg’s test and Egger’s test were applied to determine the statistical significance of publication bias.

### Statistical Analysis

Systematic review and meta-analysis were performed using RevMan 5.2. Dichotomous variables were expressed as pooled risk ratios (RR) with 95% confidence interval (CI) and continuous variables were expressed as the weighted mean difference (MD) with 95% CI. Heterogeneity across the RCTs was assessed by Chi-squared test and I^2^ statistic. If *p* < 0.05 or I^2^ > 50%, it suggested that there was significant statistical heterogeneity and the random-effect model was used to calculate the outcomes; otherwise, the fixed-effect model was considered. The Z-test was employed to verify the overall effects of ligustrazine injection as adjunctive therapy over using Western medicine alone in the treatment of ACI. Significant statistical difference was considered in this meta-analysis when *p* < 0.05.

## Results

### Study Selection

**FIGURE 1:**
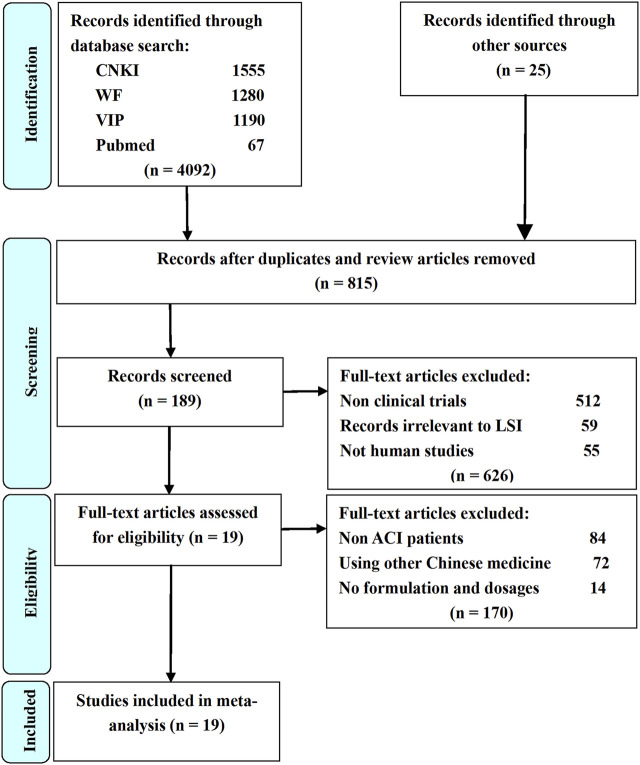
Flow diagram of study selection process. LSI is ligustrazine injection; CNKI is China National Knowledge Infrastructure; WF is Wangfang Data; VIP is Chinese Scientific Journal Database; Other sources are Medline, Chinese Biomedical Literature. Database, Cochrane Library, Embase, Google Scholar, Sino-Med, and Chinese Science Citation Database.

Study selection process and results are shown in Figure 1. A total of 4117 potential studies were initially identified from the databases based on the search strategies in the methods. Among them, 3302 studies were excluded because they were duplications, reviews, or animal experiments. Full texts of 189 studies were subjected to manual screening according to the eligibility criteria described in the methods. Finally, 19 RCTs were included for quality evaluation and meta-analysis.

### Study Characteristics

Characteristics of the included RCTs are provided in Table 1. All the 19 RCTs were published in Chinese journals (Wei, 2007; He et al., 2009; Qi, 2009; Wang, 2009; Wang et al., 2009; Chang, 2011; She et al., 2011; Xie, 2011; Xu et al., 2011; Chen and Wen, 2012; Ma, 2012; Wang, 2012; Wang et al., 2012; Lian, 2013; Zhang et al., 2013; Ma, 2014; Xia et al., 2016; Zhou, 2017; Peng, 2019). The participants in the 19 RCTs ranged from 42 to 300, with a total of 2022 ACI patients. The mean sample size of the included RCTs was 106.4 and the age of ACI patients was between 36 and 89 years old. The duration of intervention was at least 7 days, mostly 14 days. The Western medicine in the control group included ozagrel sodium injection, fibrinogenase injection, citicoline sodium injection, nimodipine, simvastatin, alteplase injection, low-molecular-weight heparins calcium injection, venorruton, clopidogrel, lumbrukinase, edaravone, mannitol injection, and aspirin. Combined with these Western medicines, the dosage of ligustrazine injection in the treatment group ranged from 80 to 240 mg per day. Seventeen RCTs employed clinical effective rate as the therapeutic indicator and five RCTs used NDS as the outcome measures. Seven RCTs reported the outcome measure fibrinogen as their therapeutic indicator. LBV was considered as the outcome measure in four RCTs and HBV was regarded as the outcome measure in six RCTs.

**TABLE 1:** Characteristics of RCTs on ligustrazine injection for ACI.

Study	Country	Year	Intervention		Sample	Follow-up (day)	Outcome measures
			Treatment group	Control group			
Chang SW	China	2011	Ligustrazine injection 80 mg/d, Ozagrel sodium injection 160 mg/d	Ozagrel sodium injection 160 mg/d	90	14	①②
Chen ZJ	China	2012	Ligustrazine injection 160 mg/d, Fibrinogenase injection 200 U/d	Fibrinogenase injection 200 U/d	100	14	①③
Lian CL	China	2013	Ligustrazine injection 120 mg/d, Citicoline sodium injection 500 mg/d, Nimodipine 120 mg/d, Aspirin 100 mg/d	Citicoline sodium injection 500 mg/d, Nimodipine 120 mg/d, Aspirin 100 mg/d	92	30	①
Ma WH	China	2014	Ligustrazine injection 160 mg/d, Citicoline sodium injection 500 mg/d, Simvastatin 20 mg/d, Aspirin 300 mg/d	Citicoline sodium injection 500 mg/d, Simvastatin 20 mg/d, Aspirin 300 mg/d	120	14	①②
Ma ZX	China	2012	Ligustrazine injection 160 mg/d, Ozagrel sodium injection 80 mg/d	Ozagrel sodium injection 80 mg/d	244	14	①③④⑤
Peng C	China	2019	Ligustrazine injection 100 mg/d, Alteplase injection 50 mg/d	Alteplase injection 50 mg/d	300	10	①②③⑤
Qi Y	China	2009	Ligustrazine injection 200 mg/d, Ozagrel sodium injection 80 mg/d	Ozagrel sodium injection 80 mg/d	100	14	①
She XB	China	2011	Ligustrazine injection 160 mg/d, Ozagrel sodium injection 80 mg/d	Ozagrel sodium injection 80 mg/d	80	14	①
Wang GL	China	2012	Ligustrazine injection 200 mg/d, Ozagrel sodium injection 80 mg/d	Ozagrel sodium injection 80 mg/d	100	14	①
Wang L	China	2009	Ligustrazine injection 200 mg/d, Ozagrel sodium injection 80 mg/d	Ozagrel sodium injection 80 mg/d	80	14	①②
Wang YC	China	2012	Ligustrazine injection 160 mg/d, Ozagrel sodium injection 80 mg/d	Ozagrel sodium injection 80 mg/d	84	14	①
Wang YL	China	2009	Ligustrazine injection 100 mg/d, Low-molecular-weight heparins calcium injection 0.4 ml/d	Low-molecular-weight heparins calcium injection 0.4 ml/d	42	7	①③
Xia XD	China	2016	Ligustrazine injection 80 mg/d, Ozagrel sodium injection 160 mg/d	Ozagrel sodium injection 160 mg/d	68	56	①③
Xie JF	China	2010	Ligustrazine injection 120 mg/d, Venorruton 500 mg/d	Venorruton 500 mg/d	84	14	①
Xu LL	China	2011	Ligustrazine injection 120 mg/d, Ozagrel sodium injection 80 mg/d, Citicoline 1 g/d, Aspirin 100 mg/d	Ozagrel sodium injection 80 mg/d, Citicoline 1 g/d, Aspirin 100 mg/d	82	14	①②③④⑤
Zhang YH	China	2013	Ligustrazine injection 240 mg/d, Aspirin 100 mg/d	Aspirin 100 mg/d	68	14	①
Zhou YF	China	2017	Ligustrazine injection 160 mg/d, Clopidogrel 75 mg/d	Clopidogrel 75 mg/d	118	14	①③④⑤
He J	China	2009	Ligustrazine injection 200 mg/d, Aspirin 100 mg/d, lumbrukinase 600, 000 U/d, Edaravone 15 ml/d	Aspirin 100 mg/d, lumbrukinase 600, 000 U/d, Edaravone 15 ml/d	60	14	④⑤
Wei N	China	2007	Ligustrazine injection 200 mg/d, Ozagrel sodium injection 80 mg/d, Nimodipine 60 mg/d, Citicoline sodium injection 150 mg, Mannitol injection 50 g/d	Ozagrel sodium injection 80 mg/d, Nimodipine 60 mg/d, Citicoline sodium injection 150 mg, Mannitol injection 50 g/d	110	14	⑤

① Clinical effective rate; ② DNS; ③ Fibrinogen; ④ LBV; ⑤ HBV.

### Risk of Bias in Individual Studies

The quality of the 19 RCTs was assessed using the Jadad scoring system (ranged from 0 to 5 scores) and the M scoring system (ranged from 1 to 7 scores), respectively. Sixteen RCTs obtained 2 scores and three RCTs obtained 3 scores according to the Jadad scale. Seven RCTs obtained 3 and ten RCTs obtained above 3 scores based on the M scale (Table 2).

**TABLE 2:** Quality of the included RCTs.

Study	M1	M2	M3	M4	M5	M score	J1	J2	J3	J score
Chang (2011)	1	1	0	1	1	4	1	0	1	2
Chen and Wen (2012)	1	1	0	1	0	3	1	0	1	2
Lian (2013)	1	1	0	1	1	4	1	0	1	2
Ma (2014)	1	1	0	1	1	4	1	0	1	2
Ma (2012)	1	1	0	1	0	3	1	0	1	2
Peng (2019)	1	2	0	1	1	5	2	0	1	3
Qi (2009)	1	1	0	1	1	4	1	0	1	2
She et al. (2011)	1	1	0	1	1	4	1	0	1	2
Wang et al. (2012)	1	1	0	1	1	4	1	0	1	2
Wang (2009)	1	1	0	1	1	4	1	0	1	2
Wang (2012)	1	1	0	1	1	4	1	0	1	2
Wang et al. (2009)	1	1	0	1	0	3	1	0	1	2
Xia et al. (2016)	1	1	0	1	0	3	1	0	1	2
Xie (2011)	1	1	0	1	1	4	1	0	1	2
Xu et al. (2011)	1	1	0	1	0	3	1	0	1	2
Zhang et al. (2013)	1	1	0	1	0	3	1	0	1	2
Zhou (2017)	1	1	0	1	0	3	1	0	1	2
He et al. (2009)	1	2	0	1	0	4	2	0	1	3
Wei (2007)	1	2	0	1	0	4	2	0	1	3

### Clinical Effective Rate

**FIGURE 2:**
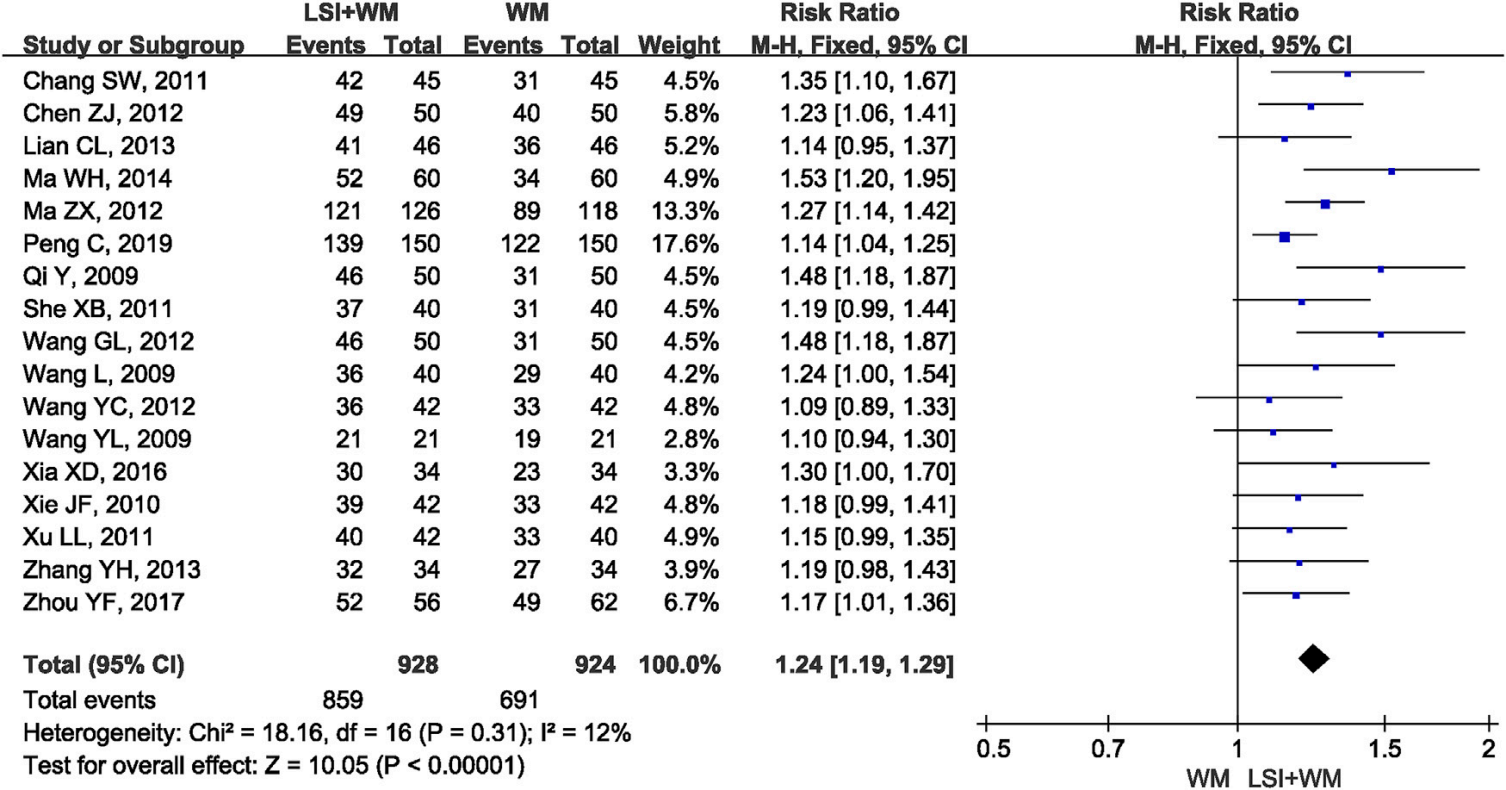
Forest plot of clinical effective rate of ligustrazine injection in treating ACI. LSI is ligustrazine injection and WM is Western medicine.

**FIGURE 3:**
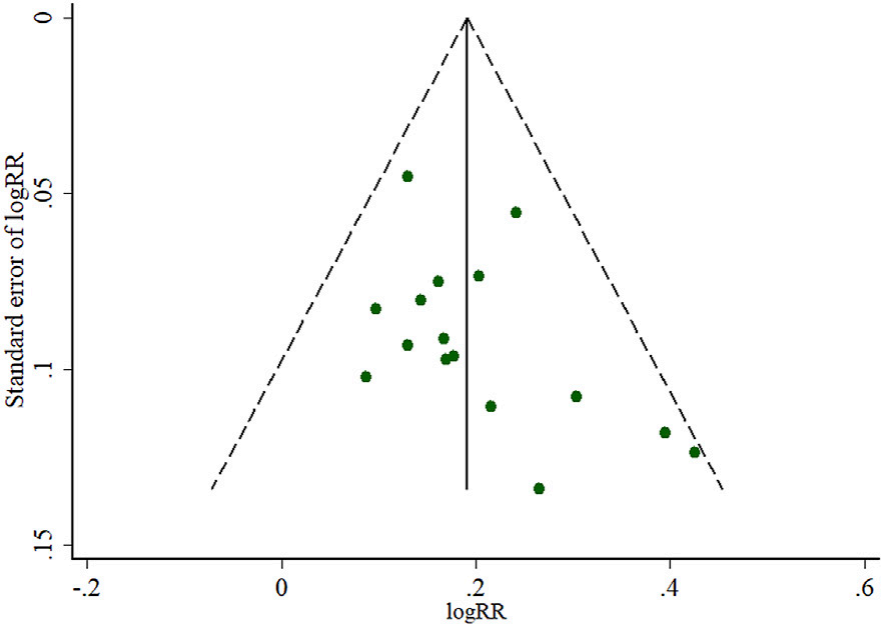
Funnel plots of the clinical effective rate of ligustrazine injection for ACI. LSI is ligustrazine injection and WM is Western medicine.

The pooled RR of clinical effective rate indicated that ligustrazine injection as adjunctive therapy was more effective than Western medicine alone in treating ACI. As shown in Figure 2, the pooled RR was 1.24 (95% CI, 1.19 to 1.29; Z = 10.05, *p* < 0.00001) with little heterogeneity (I^2^ = 12%; χ^2^ = 18.16; df = 16, *p* = 0.31) among the 17 RCTs using clinical effective rate as outcome measures. Publication bias of the primary outcome measure (clinical effective rate) was evaluated by funnel plots (Figure 3) and results showed that slight asymmetry was found in the plots. The results of Begg’s test (Z = 2.47, *p* = 0.013) and Egger’s test (t = 2.22, *p* = 0.042) suggested that publication bias was small. As shown in Table 3, sensitivity analysis was conducted to verify whether and how much the overall effect would be affected by the low quality RCTs. No significant difference was found during the gradual exclusion of lower quality of RCTs based on the M scoring system. There were slight changes (0.03 in magnitude) between the low quality (M scores ≤3) and high quality of RCTs (M scores >3). Subgroup analysis was conducted to evaluate whether the overall effect was affected by the publication date, sample size of RCTs, and dosages of ligustrazine injection. No significant difference was found in the pooled risk ratios of clinical effective rate (Table 3), which consistently demonstrated that ligustrazine injection as adjunctive therapy was apparently more effective in treating ACI.

**TABLE 3:** Sensitivity and subgroups analysis based on clinical effective rate.

	Group	No. of RCTs	No. of patients	RR	95% CI	Z	P (effect)	I^2^ (%)	χ^2^	P (het)
M score	0–5	17	1852	1.24	1.19, 1.29	10.05	<0.00001	12	18.16	0.31
	1–5	17	1852	1.24	1.19, 1.29	10.05	<0.00001	12	18.16	0.31
	2–5	17	1852	1.24	1.19, 1.29	10.05	<0.00001	12	18.16	0.31
	3–5	17	1852	1.24	1.19, 1.29	10.05	<0.00001	12	18.16	0.31
	4–5	10	1130	1.25	1.18, 1.31	7.74	<0.00001	40	15.01	0.09
	≤3	7	722	1.22	1.15, 1.29	6.46	<0.00001	0	3.10	0.80
	>3	10	1130	1.25	1.18, 1.31	7.74	<0.00001	40	15.01	0.09
Sample size	<100	10	770	1.19	1.12, 1.27	5.53	<0.00001	0	4.06	0.91
	≥100	7	1082	1.27	1.20, 1.34	8.46	<0.00001	53	12.79	0.05
Year of publication	<2013	11	1086	1.26	1.19, 1.33	8.31	<0.00001	9	10.94	0.36
	≥2013	6	766	1.21	1.13, 1.29	5.73	<0.00001	20	6.22	0.29
Dosage (mg)	≤120	7	758	1.18	1.11, 1.25	5.39	<0.00001	0	3.64	0.72
	>120	10	1094	1.28	1.21, 1.36	8.53	<0.00001	15	10.61	0.30

### NDS

**FIGURE 4:**
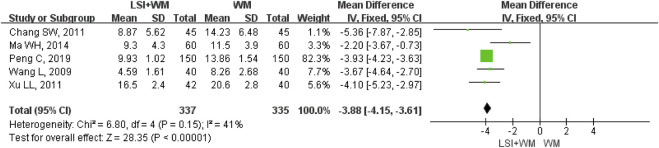
Forest plot of NDS of ligustrazine injection in treating ACI. LSI is ligustrazine injection and WM is Western medicine.

Five RCTs reported the NDS as their secondary outcome measure for ACI patients. As shown in Figure 4, ligustrazine injection as adjunctive therapy significantly reduced the NDS in ACI patients (MD = −3.88; 95% CI, −4.15 to −3.61; Z = 28.35, *p* < 0.00001) with moderate heterogeneity (I^2^ = 41%; χ^2^ = 6.80; df = 4, *p* = 0.15). As shown in Supplementary Table S3, results from the sensitivity analysis and subgroup analysis showed that no significant difference was found in the overall mean differences (MDs) of NDS. Results of Begg’s test (Z = −0.24, *p* = 1.00) and Egger’s test (t = 0.37, *p* = 0.739) suggested that there were no significant publication biases among the five RCTs.

### Fibrinogen

**FIGURE 5:**
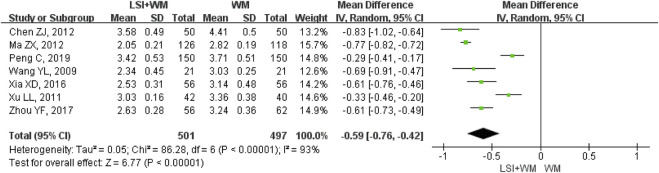
Forest plot of fibrinogen of ligustrazine injection in treating ACI. LSI is ligustrazine injection and WM is Western medicine.

Seven RCTs mentioned fibrinogen as the secondary outcome measure. As shown in Figure 5, results indicated that ligustrazine injection combined with Western medicine was superior to Western medicine alone in reducing the fibrinogen in ACI patients (MD = −0.59; 95% CI, −0.76 to −0.42; Z = 6.77, *p* < 0.00001) with high heterogeneity (I^2^ = 93%; χ^2^ = 86.28; df = 6, *p* < 0.00001). As shown in Supplementary Table S4, sensitivity analysis and subgroup analysis showed that there were little changes in the overall MDs of fibrinogen. Results from Begg’s test (Z = 0.30, *p* = 0.764) and Egger’s test (t = 1.24, *p* = 0.269) indicated that no significant publication biases were found among the included seven RCTs.

### LBV

**FIGURE 6:**
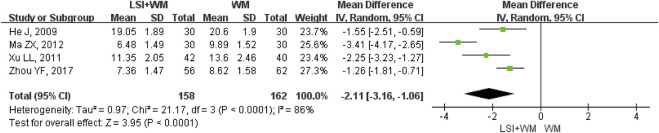
Forest plot of LBV of ligustrazine injection in treating ACI. LSI is ligustrazine injection and WM is Western medicine.

LBV was reported as the outcome measure in four RCTs. As shown in Figure 6, results suggested that ligustrazine injection as adjunctive therapy was better than Western medicine alone in reducing LBV for ACI patients (MD = −2.11; 95% CI, −3.16 to −1.06; Z = 3.95, *p* < 0.0001) with high heterogeneity (I^2^ = 86%; χ^2^ = 21.17; df = 3, *p* < 0.0001). Sensitivity analysis and subgroup analysis were performed to evaluate the overall MDs influenced by the factors of low quality RCTs, sample size and publication date. Results showed that little differences were found in the overall MDs of LBV (Supplementary Table S5). Results of Begg’s test (Z = 0.34, *p* = 0.734) and Egger’s test (t = −0.64, *p* = 0.585) demonstrated that there were no significant publication biases among the four RCTs.

### HBV

**FIGURE 7:**
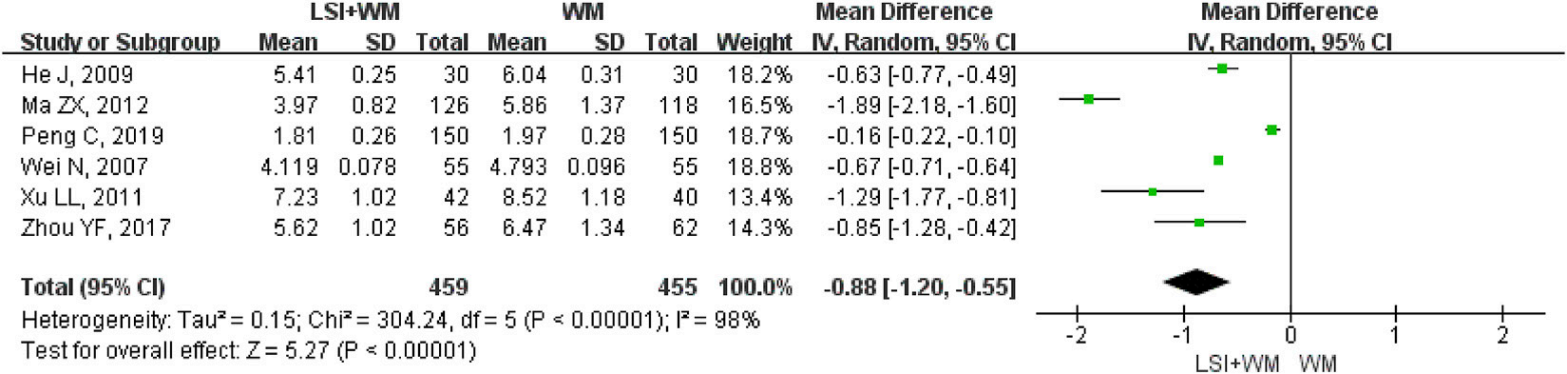
Forest plot of HBV of ligustrazine injection in treating ACI. LSI is ligustrazine injection and WM is Western medicine.

HBV was reported in six RCTs and it was selected for one of the outcome measures. As shown in Figure 7, results demonstrated that ligustrazine injection as adjunctive therapy was more effective than Western medicine alone to reduce HBV in the treatment of ACI patients (MD = −0.88; 95% CI, −1.20 to −0.55; Z = 5.27, *p* < 0.00001) with high heterogeneity (I^2^ = 98%; χ^2^ = 304.24; df = 5, *p* < 0.00001). In this meta-analysis, sensitivity analysis and subgroup analysis were conducted to verify if the overall MDs were affected by the low quality of RCTs, sample size and publication date and results suggested that little differences were found in the overall MDs of HBV (Supplementary Table S6). Results of Begg’s test (Z = 0, *p* = 1.00) and Egger’s test (t = −0.41, *p* = 0.70) indicated that no significant publication biases were found among the six RCTs.

### Adverse Reaction

Twelve RCTs indicated that the use of ligustrazine injection did not bring obvious adverse reactions for ACI patients during the intervention. The rest of the RCTs did not report adverse reactions.

## Discussion

At present, conventional therapy with Western medicines mainly includes thrombolysis, controlling cerebral edema, improving microcirculation, preventing and treating complications, the applying of neuroprotective agents restoring, blood supply to ischemic area, reducing blood viscosity, and controlling hypertension. Although these Western medicines can accelerate a patient’s recovery to a certain extent, drug side effects and resistances are accompanied with their wide application in clinical practice (CAST (Chinese Acute Stroke Trial) Collaborative Group, 1997; International Stroke Trial Collaborative Group, 1997). Besides, frequent use of Western medicines might cause motor weakness on one or both sides of the body (Li et al., 2014). Therefore, more effective agents for ACI patients are highly demanded. In past years, the role of TCM in global health care has now been accepted by more and more physicians and researchers. The increasing number of systematic review and meta-analysis on the clinical efficacy of TCM as adjunctive therapy for treating various diseases can be the best evidence (Shi et al., 2021). Numerous studies have indicated that the combination of ligustrazine injection and Western medicines is beneficial for the treatment of ACI.

Recently, modern pharmacological experimental studies showed that ligustrazine plays important roles in antithrombosis *in vivo* and the mechanism could be attributed to the inhibition of platelet aggregation and protection of endothelium (Yang et al., 2012). As a new type of calcium ion antagonist and free radical scavenger, ligustrazine can pass through the blood brain barrier. High dose of ligustrazine can significantly increase the expression of Bcl-2 and reduce the expression of p53 (Zhang and Li, 2003). Besides, ligustrazine can alleviate cerebral ischemia reperfusion injury by regulating caspase-12 gene expression, thus reducing neuronal apoptosis (Tian et al., 2014). Although a large number of pharmacological experiments have demonstrated that ligustrazine injection is an effective agent for treating ACI, a comprehensive and systematic evaluation of ligustrazine injection for the treatment of ACI is rare, based on current rigorous international standards. Ligustrazine injection has been widely used to treat ACI in China over the past decades, this famous Chinese medicine is completely unknown to most Western physicians and researchers. Therefore, this study aims to provide an internationally accessible systematic review for evaluating the effectiveness and safety of ligustrazine injection in the treatment of ACI.

### Analysis of Effectiveness

Many RCTs on ligustrazine injection combined with Western medicine for the treatment of ACI. However, the clinical efficacy of ligustrazine injection is not yet well confirmed. Although a former meta-analysis published in 2016 had evaluated the efficacy and safety of ligustrazine in the treatment of cerebral infarction, the poor methodological quality (the Jadad score of all the studies was 1 point) prevented the author from making firm conclusions (Yu et al., 2016). The lack of a subgroup and sensitive analysis also resulted in its result being more unreliable. Moreover, the former meta-analysis was not conducted according to the PRISMA guidelines. This study aims to provide a PRISMA-compliant systematic review (PRISMA Checklist was provided in Supplementary Table S7) and meta-analysis for evaluating the efficacy of ligustrazine injection as adjunctive therapy in treating ACI. Meta-analysis results of the primary outcome measure (clinical effective rate) showed that ligustrazine injection combined with Western medicine appeared to be more effective than Western medicine alone (RR = 1.24; 95% CI, 1.19–1.29). Results of the secondary outcome measures including NDS (MD = −3.88; 95% CI, −4.15 to −3.61), fibrinogen (MD = −0.59; 95% CI, −0.76 to −0.42), LBV (MD = −2.11; 95% CI, −3.16 to −1.06), and HBV (MD = −0.88; 95% CI, −1.20 to −0.55) confirmed that the therapeutic effect of ligustrazine injection as adjunctive therapy surpassed the Western medicine alone in the treatment of ACI. Moreover, the combination of ligustrazine injection did not result in adverse reactions for ACI patients.

ACI is the ischemic necrosis or cerebral softening of local brain tissues caused by the obstruction of acute local blood supply in brain tissues, ischemia, and hypoxia (Regenhardt et al., 2018). The decrease or interruption of cerebral blood flow is the main reason of ACI, which seriously damages the function of the nervous system. Clinical efficacy of ligustrazine in the remediation of neurological deficits and reduction of the fibrinogen, LBV, and HBV has been demonstrated by many pharmacological experiments. Kong et al. found that ligustrazine could promote neural progenitor cells move to the damaged area by activating the phosphatidylinositol 3-kinase pathway to achieve the protective effect on the brain (Kong et al., 2016). Whole-blood viscosity and platelet aggregation are usually used as the prognostic indicators of ischemic cerebral and myocardial diseases. Cai et al. confirmed that ligustrazine could significantly decrease whole-blood viscosity and inhibit platelet aggregation through down-regulate the expression of CXCR4 in platelets, lymphocytes, and blood red cells (Cai et al., 2014). These available evidences also consistently supported the adjunctive use of ligustrazine injection in the treatment of ACI.

### Limitations for This Review

Several potential limitations were found in this meta-analysis. First, all the included RCTs were collected from Chinese journals, restricting their worldwide attention. Second, almost all RCTs were not strictly designed based on the gold standard. Many RCTs did not clearly describe the allocation concealment and blinding, causing the potential for publication bias and overvaluation or undervaluation of the efficacy of ligustrazine injection. Third, various dosages of ligustrazine injection in the treatment groups might introduce heterogeneity for the results of the meta-analysis. Fourth, the sample size of some RCTs was relatively small and the duration of intervention was relatively short, which might cause inaccurate results for the meta-analysis. Hence, more strictly designed RCTs with higher quality, larger sample sizes, and longer duration of intervention are necessary to accurately confirm the efficacy of ligustrazine injection as adjunctive therapy for ACI.

### Implications for Further Research

The methodological quality of many RCTs was poor. Hence, future studies on ligustrazine injection should be strictly performed in accordance with the standards of reporting trials.

At present, the efficacy of ligustrazine injection as adjunctive therapy in treating ACI has not been confirmed by a PRISMA-compliant systematic review and meta-analysis. Besides, adjunctive therapies of ligustrazine injection were rarely known by most Western physicians. Therefore, we conducted a PRISMA-compliant systematic review and meta-analysis for ligustrazine injection. This meta-analysis was superior to other studies on ligustrazine injection: (1) Other Chinese medicine involved in the control or treatment group was excluded during the study selection; (2) sensitivity analysis and subgroup analysis was conducted in this meta-analysis; (3) non-RCTs were excluded in this study; and (4) this meta-analysis was strictly performed according to the PRISMA requirements.

In general, the meta-analysis results based on the 19 RCTs suggested that ligustrazine injection as adjunctive therapy could provide a better efficacy in treating ACI than Western medicine alone. Because of the low quality of the 19 RCTs, further RCTs with higher methodological quality, larger sample size, and longer duration are still recommended to validate the efficacy of ligustrazine injection.

## Conclusion

This meta-analysis suggested that ligustrazine injection as adjunctive therapy was more effective than Western medicine alone in the treatment of ACI. Ligustrazine injection combined with Western medicine was recommended to improve the total effective rate, remedy neurological deficits and reduce the fibrinogen, LBV, and HBV for ACI patients. There were no serious adverse reactions when using ligustrazine injection. However, more evidence is needed to accurately validate the efficacy of ligustrazine injection as the adjunctive therapy in treating ACI since the methodological quality of some RCTs was low.

## Data Availability

The original contributions presented in the study are included in the article/Supplementary Material. Further inquiries can be directed to the corresponding authors.

## Abbreviations

ACI, acute cerebral infarction; CI, confidence interval; CNKI, China national knowledge infrastructure; HBV, high shear blood viscosity; LBV, low shear blood viscosity; LSI, ligustrazine injection; MD, mean difference; NDS, neurological deficit score; RCTs, randomized controlled trials; RR, relative risk; rt-PA, recombinant tissue plasminogen activator; TCM, traditional Chinese medicine; UK, urokinase; VIP, China Science and Technology Journal Database; WM, western medicine.
